# Dual-Antigen COVID-19 Vaccine Subcutaneous Prime Delivery With Oral Boosts Protects NHP Against SARS-CoV-2 Challenge

**DOI:** 10.3389/fimmu.2021.729837

**Published:** 2021-09-16

**Authors:** Elizabeth Gabitzsch, Jeffrey T. Safrit, Mohit Verma, Adrian Rice, Peter Sieling, Lise Zakin, Annie Shin, Brett Morimoto, Helty Adisetiyo, Raymond Wong, Ashish Bezawada, Kyle Dinkins, Joseph Balint, Victor Peykov, Hermes Garban, Philip Liu, Andrew Bacon, Pete Bone, Jeff Drew, Daniel C. Sanford, Patricia Spilman, Lennie Sender, Shahrooz Rabizadeh, Kayvan Niazi, Patrick Soon-Shiong

**Affiliations:** ^1^ImmunityBio, Inc., Culver City, CA, United States; ^2^NantKwest, Inc., Culver City, CA, United States; ^3^IosBio, Burgess Hill, United Kingdom; ^4^Battelle Biomedical Research Center, Columbus, OH, United States

**Keywords:** non-human primate (NHP), vaccine, dual antigen, COVID-19, SARS-CoV-2 challenge, protection, lung, nasal passages

## Abstract

We have developed a dual-antigen COVID-19 vaccine incorporating genes for a modified SARS-CoV-2 spike protein (S-Fusion) and the viral nucleocapsid (N) protein with an Enhanced T-cell Stimulation Domain (N-ETSD) to increase the potential for MHC class II responses. The vaccine antigens are delivered by a human adenovirus serotype 5 platform, hAd5 [E1-, E2b-, E3-], previously demonstrated to be effective in the presence of Ad immunity. Vaccination of rhesus macaques with the hAd5 S-Fusion + N-ETSD vaccine by subcutaneous prime injection followed by two oral boosts elicited neutralizing anti-S IgG and T helper cell 1-biased T-cell responses to both S and N that protected the upper and lower respiratory tracts from high titer (1 x 10^6^ TCID_50_) SARS-CoV-2 challenge. Notably, viral replication was inhibited within 24 hours of challenge in both lung and nasal passages, becoming undetectable within 7 days post-challenge.

## Introduction

**Figure 1:**
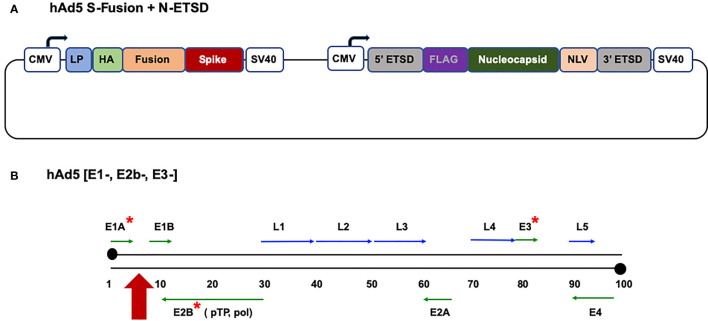
*The hAd5 S-Fusion + N-ETSD vaccine and the hAd5 [E1-, E2b-, E3-] platform*. **(A)** The dual-antigen vaccine delivered by the hAd5 [E1-, E2b-, E3-] platform comprises both the spike (S) and nucleocapsid (N) SARS-CoV-2 antigens. The S construct starts with the S leader peptide (LP), followed by human influenza hemagglutinin (HA), a ‘fusion’ linker, and the S sequence. The N construct is bracketed by 5’ and 3’ ETSD sequences and has FLAG, N, and NLV sequences. Expression of both antigens is under control of a cytomegalovirus (CMV) promoter and both end with C-terminal SV40 poly-A sequences. **(B)** The human adenovirus serotype 5 vaccine platform with E1, E2b, and E3 regions deleted (*) is shown. The vaccine construct is inserted in the E1 regions (red arrow).

To address the ongoing COVID-19 pandemic (1), particularly in the face of viral evolution and evidence of viral variant resistance to antibodies and convalescent plasma (2–5), we have developed a vaccine anticipated to protect individuals from SARS-CoV-2 that has the potential to not only elicit robust humoral responses but also activate T cells. The dual-antigen vaccine (**Figure 1A**) comprises the SARS-CoV-2 spike protein fused to a signal sequence (S-Fusion) that, as predicted based on reports for similar sequences (6, 7), in our previous *in vitro* studies enhances cell-surface expression of the spike receptor binding domain (S RBD) as compared to S wildtype (8, 9). The vaccine also delivers the viral nucleocapsid (N) protein with an Enhanced T-cell Stimulation Domain (N-ETSD) that directs N to the endo/lysosomal subcellular compartment as confirmed by immunohistochemistry (10). Compared to N wild type, N-ETSD induced higher levels of interferon-γ in CD4+ T cells from 2 of 3 individuals previously infected with SARS-CoV-2 in Sieling et al. (10), consistent with the hypothesis that endosomal targeting enhances MHC class II restricted T cell responses (11–13).

The SARS-CoV-2 vaccine antigens are delivered by a recombinant human adenovirus serotype 5 (hAd5) [E1-, E2b-, E3-] vector platform (**Figure 1B**) we previously developed to rapidly generate vaccines against multiple agents (14–16). The hAd5 platform has unique deletions in the early 1 (E1), early 2b (E2b) and early 3 (E3) regions [hAd5 (E1-, E2b-, E3-)], which distinguishes it from other adenoviral vaccine platform technologies under development (17–19), and allows it to be effective in the presence of pre-existing adenovirus immunity (14, 20–22). The deletions in the E2b region DNA polymerase and preterminal proteins result in a decrease of late gene expression, including the Ad5 fiber (19), which results in a marked reduction in host inflammatory responses to the vector (23). Deletion of E2b also reduces the theoretically increased risk of HIV-1 infection associated with use of an Ad5 vector in HIV-exposed populations (24). We have utilized this platform to produce vaccines against viral antigens such as Influenza, HIV-1, H1N1, and Lassa fever that have elicited both humoral and cell mediated immunity (21, 25–28).

The overwhelming majority of other SARS-CoV-2 vaccines in development target only the wildtype S antigen and are expected to elicit SARS-CoV-2 neutralizing antibody responses. In the development of our vaccine, the addition of N in particular was predicted to afford a greater opportunity for T cell responses (29–32). In a study of SARS-CoV-2 convalescent patients, virus-specific T cells were detected in most patients, including asymptomatic individuals, even those with undetectable antibody responses (32). In the rhesus macaque model, McMahan et al. demonstrated that depletion of CD8+ T cells decreased protection conferred by previous SARS-CoV-2 infection, suggesting cell-mediated protection may be vital in the presence of declining levels of protective antibody titers (33). Relevant to increasing the potential for protection in the face of emerging variants, T-cell responses have been reported to be less vulnerable to loss of efficacy against variants than humoral responses (4, 34, 35).

While an early report generated some concern about an association of an N-based vaccine and lung pathology in a mouse model (36), many subsequent reports dispelled this fear and even associate generation of anti-N antibodies with protection of the lung from pathology after SARS-CoV-2 challenge (37) and the prevention of spread of viral infection to distal organs (38).

In our preliminary studies of the hAd5 S-Fusion + N-ETSD vaccine in a murine model, we demonstrated that the vaccine not only elicits T helper cell 1 (Th1)-biased antibody responses to both S and N, it activates T cells (8, 9). We have also confirmed that the vaccine antigens re-capitulate T-cell activation prompted by natural SARS-CoV-2 infection by demonstrating activation of COVID-19 convalescent patient CD4+ and CD8+ T cells upon exposure to homologous monocyte-derived dendritic cells (MoDCs) that had been transduced with the vaccine (10). This convalescent T-cell response to vaccine antigens suggests that, conversely, hAd5 S-Fusion + N-ETSD vaccination will generate T cells that will recognize SARS-CoV-2 antigens upon viral infection with the potential to protect the vaccinated individual from disease.

We have further reported preliminary data from Phase 1b testing of the vaccine, showing that not only does a single prime SC vaccination elicit T-cell responses to both S and N peptides, these responses were maintained against S peptides with the mutations found in the B.1.351, B.1.1.7, B.1.492 and P.1 variants (10).

Here, we tested the ability of the hAd5 S-Fusion + N-ETSD vaccine to provide protection against high titer (1 x 10^6^ TCID_50_) SARS-CoV-2 infection in the rhesus macaque non-human primate (NHP) model. We compared subcutaneous (SC) prime delivery followed by a Day 14 SC boost and a Day 28 oral boost (SC > SC > Oral) to an SC prime with two oral boosts (SC > Oral > Oral) given at the same intervals. The boost interval of 2 weeks was chosen so that we could collect data on the effects of boosts on humoral and T-cell activation. Here, and in our current clinical trials, we have utilized the SC rather than the intramuscular route because the SC delivery both affords an opportunity to recruit dendritic cells (39, 40) and is the delivery route we have used successfully in our ongoing development of vaccines against tumor antigens.

Both dosing regimens elicited similar production of virus-neutralizing anti-S IgG and activated T cells that displayed Th1-biased responses to both S and N peptides. In the challenge study, SARS-CoV-2 virus was more rapidly cleared from the both the nasal passages and lungs of vaccinated NHP following either regimen as compared to unvaccinated controls. Further, a microneutralization assay demonstrated a continued increase in SARS-CoV-2 neutralizing antibodies in the nasal passages of vaccinated, but not unvaccinated, NHP in the 14 days after challenge. Of note, viral titers dropped within the first 24 hours of challenge and SARS-CoV-2 subgenomic RNA (sgRNA) was below the level of detection (LOD) in the nasal passages of 9 of 10 vaccinated NHP by Day 5 post-challenge and in all by Day 7; in bronchoalveolar lavage (BAL) samples, sgRNA was below the LOD in 4 of 10 vaccinated NHP by Day 5 and all by Day 7 post-challenge. In both nasal passages and BAL samples of placebo NHP, sgRNA was still detectable at Day 7 post-challenge.

## Methods

### The hAd5 S-Fusion + N-ETSD Vaccine

To generate the hAd5 S-Fusion + N-ETSD vaccine, we cloned the S leader peptide, a human influenza hemagglutinin (HA) tag, a proprietary ‘fusion’ linker, and the wildtype S sequence [GenBank accession number MN908947] to the transmembrane domain into the hAd5 [E1-, E2b-, E3-] platform (**Figure 1B**). The sequences used (with the exception of ‘fusion’) are shown in the **Supplementary Materials**. The SARS-CoV-2 S protein is found on the viral surface and its receptor binding domain (RBD) interacts with the host angiotensin-converting enzyme 2 (ACE2) and gains entry to the host cell to initiate infection (41). Antibodies against the S RBD are neutralizing, preventing this first step in infection (42–44).

We also inserted a wildtype nucleocapsid (N) sequence [accession number MN908947] with both a 5’ and 3’ proprietary Enhanced T-cell Stimulation Domain (ETSD) to direct translated N to the endosomal/lysosomal pathway (10, 11). The sequences used (with the exception of ETSD) are shown in the Supplementary Materials. The N protein is found in the interior of the virus and is highly conserved and antigenic (45, 46). N also plays an important role in T-cell responses (47, 48). Note the term fusion does not apply to expression of the two antigens, but rather the modification of S, as described above.

The powerful cytomegalovirus (CMV) promoter (49, 50) drives expression in the hAd5 construct and each antigen sequence is followed by a C-terminal SV40 poly-A sequence, as shown in **Figure 1A**. To avoid the risk of recombination events during manufacturing, passages are kept to a minimum and, for GMP manufacture, release testing of the Drug product includes insert sequencing to confirm the correct insert is present and immunoblot is used to confirm the presence of both antigens.

### The NHP Study

The study, performed at Battelle Biomedical Research Center (Columbus, Ohio), was sponsored by the Biomedical Advanced Research & Development Authority (BARDA), Office of the Assistant Secretary for Preparedness and Response (ASPR), Department of Health and Human Services (DHHS) and the National Institutes of Health/National Institute for Allergy and Infectious Diseases (NIH/NIAID) (Washington, DC). Battelle is a Public Health Service (PHS) Animal Welfare Assurance approved facility. The study protocol was approved by the Institutional Animal Care and Use Committee (IACUC). All aspects of the animal study protocol were designed to minimize stress in the animals.

### Dosing and Sample Collection

A total of 12 naïve rhesus macaques weighing >/= 2.5 Kg and being >2.5 years of age were used in the study. All rhesus macaques were tested and confirmed negative within 45 days of receipt for Mycobacterium tuberculosis, simian immunodeficiency virus (SIV), simian T-lymphotropic virus-I (STLV-1), simian retroviruses 1 and 2 (SRV-1 and SRV-2) *via* PCR, Macacine herpesvirus I (Herpes B virus), and Trypanosoma cruzi (ELISA and PCR).

**Figure 2:**
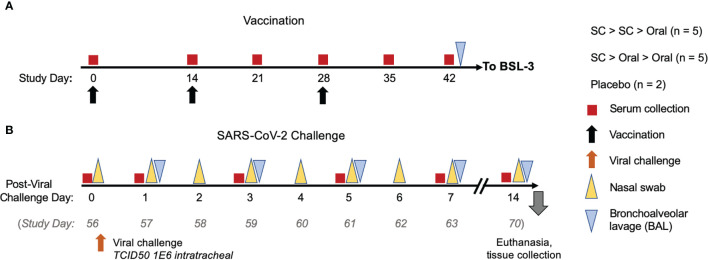
*NHP study design*. **(A)** NHP were vaccinated either by subcutaneous (SC) injection of 1 x 10^11^ VP hAd5 S-Fusion + N-ETSD on Study Days 0 and 14, with an oral boost of 1 x 10^10^ IU on Study Day 28 (n = 5) or received one SC prime injection and two oral boosts (n = 5). Control NHP received placebo by an SC > SC > Oral regimen (n = 2). Sera were collected as indicated (red boxes) throughout the study. **(B)** On Study Day 42, NHP were transferred to a BSL-3 facility for viral challenge (orange arrow) on *Study Day 56* (described as Challenge Day 0 for post-challenge analyses here) with 1x10^6^ TCID_50_ VP of SARS-CoV-2 intranasally and intratracheally. Nasal samples (yellow triangles) and bronchoalveolar lavage (BAL) samples (gray triangles) were collected as indicated. Animals were euthanized 14 days after challenge and tissues collected for pathology.

We compared two SC injections administered in the center of the back just caudal to the scapular region of 1 x 10^11^ vaccine particles (VP) of hAd5 S-Fusion + N-ETSD on Days 0 and 14 followed by an oral capsule 1x 10^10^ infectious units (IU) of hAd5 S-Fusion + N-ETSD delivered *via* a feeding tube after a minimum of 4 hours of fasting on Day 28 (SC > SC > Oral, Group 1) to one prime SC injection and two oral boost doses with the same dosages and timing (SC > Oral > Oral, Group 2), as shown in **Figure 2**. The VP to IU ratio, used as a quality limit, for the lot used for SC injection was 28:1. SC dosing is based on VPs to control for the number of virus particles (infectious and non-infectious) introduced by that route in that route; for the oral route, the dosing metric is IU because the material is not purified thus VP determination is not possible.

Vaccination Group 1 (SC > SC > Oral comprised 3 male and 2 female, Group 2 (SC > Oral > Oral) 2 male and 3 female, and Group 3 (placebo) 1 male and 1 female randomized NHP.

On Day 42, NHPs were transferred to a BSL-3 facility and on Day 56 – what we will refer to as challenge Day 0 in Results - they were challenged *via* the intratracheal (0.5 mL) and intranasal (0.25 mL per nares) routes with a total dose of approximately 1 x10^6^ TCID_50_ SARS-CoV-2 strain USA-WA1/2020. Nasal and oropharyngeal swabs were collected daily from challenge Day 0 (prior to challenge) through 7 days post-challenge and again 14 days post-challenge. In addition, bronchoalveolar lavages (BALs) were performed on challenge Days 1, 3, 5, and 7.

### Clinical Signs

NHP in all groups were observed twice daily from study Day -7 until the end of the study on challenge Day 14 for clinical signs, including but not limited to anorexia (weights were taken), hunched posture, lethargy, respiratory distress, activity (recumbent, weak, or unresponsive), convulsions, and other abnormal clinical observations. Blood was collected from a femoral artery or vein, saphenous vein, or appropriate vessel of anesthetized animals at baseline, and study Days 14, 21, 28, 35, 42, 56/challenge Days 0, 1, 3, 5, 7, and 14 (End Study). Collected blood was used for clinical chemistry and hematological analyses as well as isolation of PBMCs. Body weights are shown in **Supplementary Figure S1**, hematology in **Supplementary Table S1** and clinical chemistry in **Supplementary Table S2**.

### Statistical Analysis

For comparison of animals in groups, one-way ANOVA was used with Dunnett’s *post-hoc* comparison of vaccinated groups to the placebo control. All statistical analysis was performed using GraphPad Prism 9 software.

### ELISA for Anti-Spike IgG

IgG against recombinant spike protein in NHP sera or plasma was determined using an Enzyme-Linked ImmunoSorbent Assay (ELISA) wherein 96 well EIA/RIA plates (ThermoFisher, Cat# 07-200-642) were coated with 50 μL/well by a 1 μg/mL solution of purified recombinant SARS-CoV-2-derived Spike protein (S-Fusion; ImmunityBio, Inc.) suspended in coating buffer (0.05 M carbonate-bicarbonate, pH 9.6) and incubated overnight at 4°C. Plates were washed three times with 150 μL of TPBS solution (PBS + 0.05% Tween 20) then 100 μL/well of blocking solution (2% non-fat milk in TPBS) was added and incubated for 1 hour at room temperature (RT). Plasma and serum samples were heat-inactivated at 56°C for 1 hour before use and 1:50 dilutions were prepared in 1% non-fat milk (NFM) in TPBS. Plates were washed as described above and 50 μL/well of each dilution was added to the plate and incubated 1 hour at RT. Plates were washed three times with 200 μL of TPBS before addition of 50 μL/well of a 1:6K dilution of HRP-conjugated, cross-absorbed goat anti-monkey IgG (H+L) secondary antibody (ThermoFisher, Cat# PA1-84631) in 1% NFM/TPBS and incubation for 1 hour at RT.

Plates were then washed three times with 200 μL of TPBS and 50 μL of 3,3’,5,5’-tetramethylbenzidine (TMB) substrate (VWR, Cat# 100359-156) was added to each well and incubated at RT for 10 minutes. The reaction was stopped by addition of 50 μL/well of 1N sulfuric acid (H_2_SO_4_). The optical density (OD) at 450 nm was measured using a Synergy 2 plate reader (BioTek Instruments, Inc) and data is analyzed using Prism 8 (GraphPad Software, LLC).

### cPass™ Surrogate Assay for Determination of the Presence of Neutralizing Antibodies

The presence of neutralizing, anti-spike antibodies in sera from all NHP was determined by assay of sera collected on Days 0, 14, 21, 28, 35 and 42 using the surrogate virus neutralization assay, cPass™ (51). The assay is based on inhibition of binding of the spike receptor binding domain (RBD) to its human host receptor (in the assay, recombinant) angiotensin converting enzyme 2 (ACE2), with inhibition above 30% being correlated with a level of SARS-CoV-2 neutralizing anti-S antibodies that correlates with neutralization as detected in the conventional live virus assay. The assay is both species- and antibody isotype- independent. As described by Tan (51), their surrogate assay is as specific as the conventional assay but more sensitive, and it correlates better with the conventional assay than pseudovirus assays; they note it detects ‘genuine’ neutralizing antibodies (nAbs) and, that while not all nAbs are RBD binders, RBD binders are immunodominant during infection (52, 53). All sera samples were diluted 1:30.

### ELISpot for Assessment of Cytokine Secretion

ELISpot assays were used to detect cytokines secreted by fresh peripheral blood mononuclear cells (PBMCs) isolated from the blood of NHP study animals. PBMCs were isolated from whole blood by standard density gradient centrifugation and frozen in liquid nitrogen until use. PBMCs were thawed and re-suspended in RPMI 10% human AB serum, then pulsed with 2 µg/ml of SARS-CoV-2 S or N peptide pools (JPT Peptide Technologies catalogue # PM-WCPV-S and PM-WCPV-NCAP-1 respectively). Negative and positive controls were cells cultured with media alone or phorbol myristate acetate (PMA, 50 ng/ml) and ionomycin (1 µg/ml), respectively. For IFN-γ assessment, PBMCs were cultured with peptides for 17 hours at 37°C in a microtiter plate (Millipore catalogue # MAIPS4510) containing an immobilized primary antibody to capture NHP-specific IFN-γ (MabTech catalogue # 3421M-3-1000). IFN-γ was detected by a secondary antibody to human IFN-γ conjugated to biotin (MabTech catalogue # 3420-6-250). A streptavidin/horseradish peroxidase conjugate (Thermo Fisher catalogue # 21126) was used detect the biotin-conjugated secondary antibody. IFN-γ spot development was completed using a peroxidase substrate kit (Vector Labs catalogue # SK-4200). The number of spots per well (3.5 x 10^5^ PBMCs) was counted using an ELISpot plate reader. IL-4 was assessed using a commercial ELISpot kit (MabTech catalogue # 3410-APW-2) using the manufacturer’s instructions. Numbers for graphing were adjusted to spot-forming cells per 10^6^ PBMCs.

### Determination of Viral Load and Viral Replication Post-Challenge

RT-qPCR assays were performed to quantify total SARS-CoV-2 RNA copies including genomic RNA using the nucleocapsid protein gene as a target or subgenomic RNA copies that are replication intermediates of the virus using the envelope protein (E) gene as a target. These assays were performed to quantify viral loads following SARS-CoV-2 challenge. RNA was isolated from swabs and bronchioalveolar lavage fluid using the Indispin QIAcube HT Pathogen Kit (Indical Bioscience, Germany) on the QIAcube HT instrument (Qiagen, Germany). The isolated RNA was then evaluated in RT-qPCR using the TaqMan Fast Virus 1-step Master Mix (Thermo Fisher Scientific) on a QuantStudio Flex 6 Real-Time PCR System (Applied Biosystems; Foster City, CA). The primers and probe for total SARS-CoV-2 RNA quantitation were specific to the nucleocapsid protein gene, corresponding to the N1 sequences from the Centers for Disease Control and Prevention (CDC) 2019-Novel Coronavirus (2019-nCoV) Real-Time RT-PCR Diagnostic Panel (https://www.cdc.gov/coronavirus/2019-ncov/lab/rt-pcr-panel-primer-probes.html) except that the probe quencher was modified to Non-Fluorescent Quencher-Minor Groove Binder (NFQ-MGB) (Thermo Fisher Scientific). The primers and probe for the subgenomic RNA quantitation were specific to the E gene subgenomic RNA (Integrated DNA Technologies, Iowa) (54). A standard curve comprised of synthetic RNA containing the corresponding target sequence from SARS-CoV-2 isolate WA1 sequence (GenBank Accession Number MN985325.1) (Bio-Synthesis, Inc.; Lewisville, TX) was included on each PCR plate for absolute quantitation of SARS-CoV-2 RNA copies in each sample. Thermocycling conditions were: Stage 1 - 50°C for 5 min for one cycle; Stage 2 - 95°C for 20 sec for one cycle; Stage 3 - 95°C for 3 sec and 60°C for 30 sec for 40 cycles. Data analysis was performed using the QuantStudio 6 software-generated values (total copies per well of each sample) and additional calculations to determine SARS-CoV-2 RNA or subgenomic RNA copies per mL of fluid.

### Microneutralization Assay (MNA)

The neutralizing antibody titer in hAd5 S-Fusion + N-ETSD vaccinated and placebo NHP sera were measured using a microneutralization assay carried out in the BSL-4. In brief, the serum samples were heat-inactivated at 56°C for 90 min, serially diluted two-fold, and pre-incubated with SARS-CoV-2 stock at 37°C for 1 hour. The virus/serum mixture was added to 90-100% confluent monolayer Vero E6 cells (BEI, Cat. No. NR-596) in 96-well plates and incubated for 2 days at 37°C with 5% CO_2_. The virus-containing medium was then replaced with 80% acetone for cell fixation. Plates were incubated with an anti-nucleocapsid protein primary antibody cocktail (clones HM1056 and HM1057; EastCoast Bio, North Berwick, ME) for 60 minutes at 37°C. The plates were washed and the secondary antibody (goat anti-mouse IgG Horse Radish Peroxidase (HRP) conjugate; Fitzgerald, North Acton, MA) was added to the wells and the plates were incubated for 60 minutes at 37°C (Battelle Memorial Institute, Patent Number 63/041,551 Pending, 2020). After the plates were washed, the substrate was added and the plates were incubated at 37°C. Stop solution was added and the plates were read for optical density at 405 nm wavelength. Neutralizing activity is defined as at least 50% reduction in signal from the virus only (VC) wells relative to cells control (CC) wells following the formula [(average VC –average CC)/2] + average CC. The median neutralizing titer (MN50) was calculated using Spearman-Kärber analysis method (55).

## Results

### Clinical Signs, Hematology, and Clinical Chemistry

No clinical signs were noted during the twice daily observations for signs of toxicity due to vaccination and no animals died during the two weeks after one subcutaneous immunization of 1x 10^11^ vaccine particles (VP) or a week after an oral booster of 1x10^10^ IU of hAd5-S-Fusion+N-ETSD. In addition, no gross pathological effects or adverse events were observed and there were no notable changes in body weight (**Supplementary Figure S1**). Lastly, hematology and clinical chemistry revealed no abnormalities as a result of vaccination (**Supplementary Tables S1** and **S2**).

### hAd5 S-Fusion + N-ETSD SC > SC > Oral and SC > Oral > Oral Vaccination Elicit Similar Levels of Neutralizing Anti-S Antibodies

**Figure 3:**
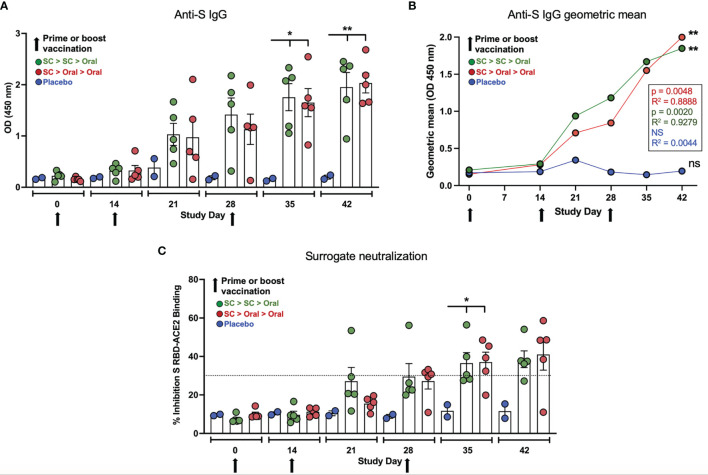
*Anti-spike (S) IgG and neutralizing capability*. **(A)** Anti-S IgG levels (ELISA; OD 450 nm) at Days 0, 14, 21, 28, 35 and 42 are shown for placebo, SC > SC > Oral and SC > Oral > Oral NHP groups. Anti-S IgG levels in both groups of vaccinated NHP were significantly higher than placebo control on Days 35 and 42 (Day 35 p = 0.0134 and = 0.0192; and Day 42 p = 0.0046 and = 0.0035 for SC > SC > Oral and SC > Oral > Oral *versus* control, respectively). **(B)** The geometric means for anti-S IgG for each group are shown. Both vaccinated groups were significantly higher than the placebo controls; p and R^2^ values are shown. **(C)** Percent inhibition in the surrogate assay where ≥ 30% (dashed line) correlates with live virus neutralization. Inhibition was significantly greater for vaccinated groups on Day 35 - p = 0.0444 and = 0.0404 - with a trend to greater inhibition on Day 42 - p = 0.0732 and = 0.0520 – both SC > SC > Oral and SC > Oral > Oral, respectively. Statistical analysis performed using one-way ANOVA with Dunnett’s *post-hoc* comparison of vaccinated groups with placebo; where *p < 0.05, **p ≤ 0.01, and ns, not significant. Data graphed as the mean and SEM.

Both SC > SC > Oral and SC > Oral > Oral vaccinated NHP produced anti-S IgG that increased after both the Day 14 and 28 boosts (**Figures 3A, B**). Anti-S IgG levels for both groups were significantly higher than placebo controls on Days 35 and 42 (**Figure 3A**). By Day 35, sera from 3 of 5 SC > SC > Oral and 4 of 5 SC > Oral > Oral vaccinated NHP demonstrated inhibition in the surrogate neutralization cPass assay that assesses the inhibition of S RBD binding to recombinant angiotensin-converting enzyme 2 (ACE2); inhibition of 30% or greater in the assay is correlated with live virus neutralization (51). By Day 42, neutralizing antibodies were detected in 8 of 10 vaccinated NHP (**Figure 3C**). The vaccinated NHP not surpassing 30% showed inhibition above 20% in the assay, whereas as inhibition was well below 20% for unvaccinated controls.

### SC > Oral > Oral Vaccination Was as Effective at Activating T Cells as SC > SC > Oral Vaccination

**Figure 4:**
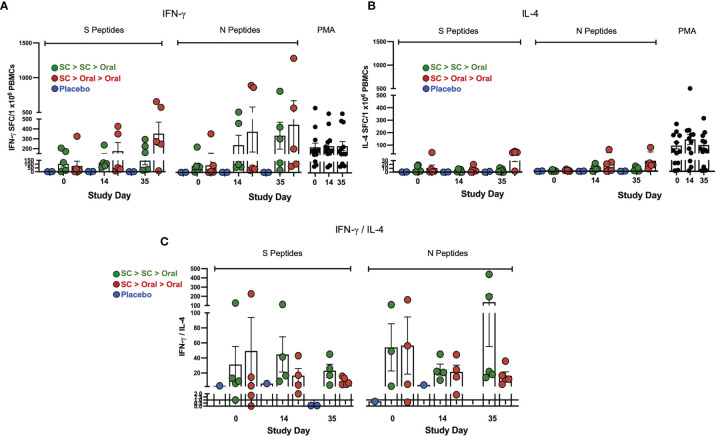
*Interferon-*γ* (IFN-*γ*) and interleukin-4 (IL-4) secretion by T cells*. **(A)** IFN-*γ*) and **(B)** IL-4 secretion by T cells in response to S and N peptides in ELISpot is shown. The PMA positive control is at right on each graph. **(C)** IFN-*γ*/IL-4 ratios greater than 1 (line) suggest T helper cell 1 (Th1) bias of T-cell responses; ratios for samples that gave IL-4 values of zero are not shown. Statistical analysis performed using one-way ANOVA with Dunnett’s *post-hoc* comparison of vaccinated groups with placebo (no significant differences were observed due to individual variation). Data graphed as the mean and SEM.

Interferon-γ (IFN-γ) secretion by T cells from vaccinated NHP was detected in response to both S1 + S2 and N peptide pools (**Figure 4A**). Responses were greater overall to N but the differences were not significant due to individual variation. The mean values were higher for the SC > Oral > Oral group as compared to SC > SC > Oral. All interleukin-4 (IL-4) responses were very low (**Figure 4B**), therefore the IFN-γ/IL-4 ratios (**Figure 4C**) were greater than 1, with one exception – an NHP with very low responses to both S and N. An IFN-γ/IL-4 greater than 1 suggests Th1 bias for T-cell responses in the vaccinated NHP.

### SC > Oral > Oral Was as Efficacious at Reducing Viral Load in Nasal Passages and Lung After SARS-CoV-2 Challenge as SC > SC > Oral

**Figure 5:**
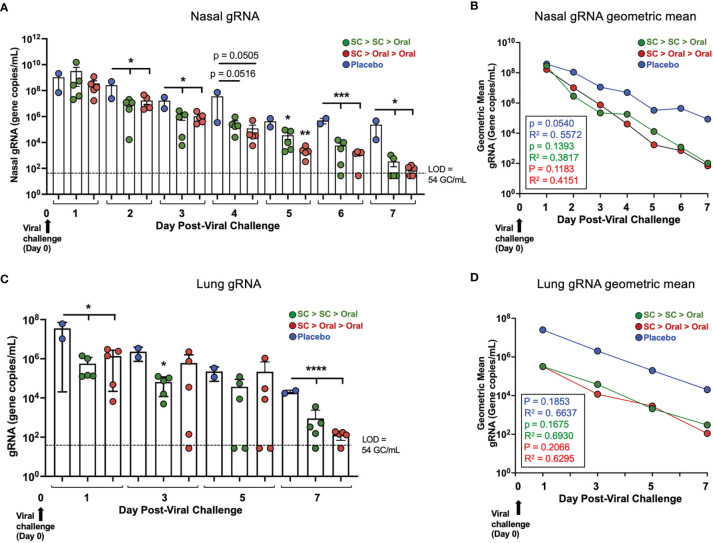
*Viral load (gRNA) in nasal passages and lung of vaccinated NHP post-challenge*. **(A)** Individual viral gRNA and **(B)** the geometric mean for nasal swab samples; and **(C)** gRNA and **(D)** the geometric mean for bronchoalveolar lavage (BAL) samples. The p and R^2^ values are shown in **(B, D)**. SARS-CoV-2 challenge was on Day 0 (Study Day 56; black arrows). The level of detection (LOD; dashed line) was 54 gene copies/mL (GC/mL) for gRNA. For values below the LOD, half the LOD value (27 GC/mL) was used for graphing of individual values and calculation of the geometric mean. Statistics performed using One-Way ANOVA with Dunnett’s *post-hoc* comparison of vaccinated groups with placebo; where *p < 0.05, **p ≤ 0.01, ***p ≤ 0.001, and ****p ≤ 0.0001. P values presented in **Supplementary Table S7**. Data graphed as the mean and SEM in **(A, C)**.

RT-qPCR analysis of genomic RNA (gRNA) was performed on nasal swab and bronchoalveolar lavage (BAL) samples to determine the amount of virus present. In both vaccinated groups 2 days after challenge, SARS-CoV-2 gRNA in the nasal swab samples was significantly decreased as compared to placebo control NHP (**Figures 5A, B**, **Supplementary Table S3**). The decreases of gRNA in vaccinated NHP continued to be greater than in the placebo groups on days 3, 5, 6 and 7 (with a trend to show greater decreases on Day 4), with viral gRNA diminishing to levels that were very low or below the level of detection (LOD) in all vaccinated animals by 7 days after challenge. Placebo controls had moderate to high levels (range 2E+09 – 8.4E+03 gene copies/mL) of SARS-CoV-2 present in nasal swab samples for the duration of the study.

In the lungs bronchoalveolar lavage, (BAL) of vaccinated NHP, gRNA also decreased rapidly, with gRNA for vaccinated NHP being significantly lower than for placebo NHP on Day 1 post-challenge and lower with high significance by Day 7 post-challenge (**Figure 5C**). The geometric mean reflects a ~2 log decrease in NHP vaccinated by either the SC > SC > Oral or SC > Oral > Oral regimens compared to placebo NHP just one day after challenge (**Figure 5D**, **Supplementary Table S4**), a difference that continued up to Day 7.

### SC > Oral > Oral Was as Efficacious at Reducing Replicating Virus in Nasal Passages and Lung After SARS-CoV-2 Challenge as SC > SC > Oral

**Figure 6:**
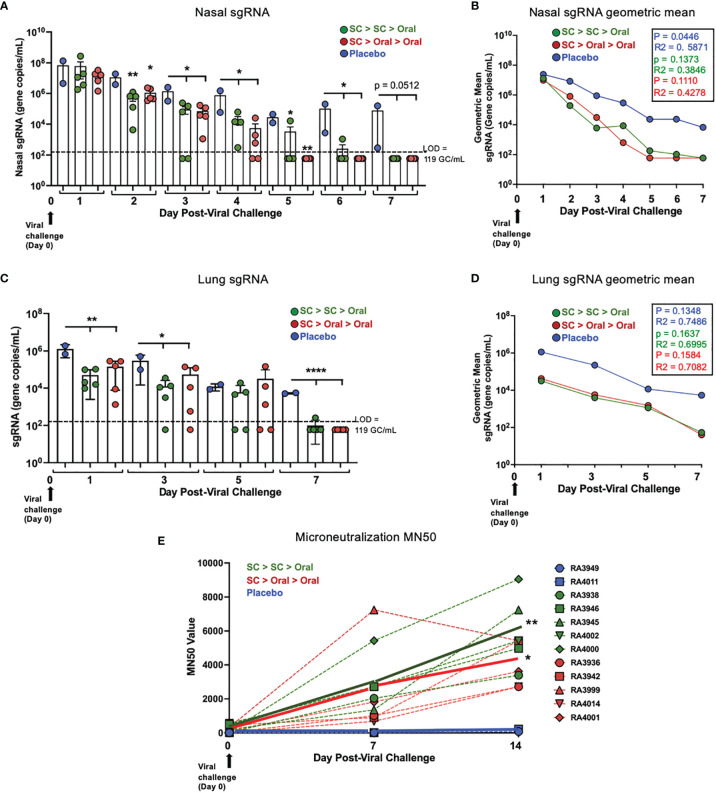
*Nasal and lung sgRNA and sera neutralization capability post-challenge*. **(A)** Individual viral sgRNA and **(B)** the geometric mean for nasal samples; and **(C)** individual viral sgRNA and **(D)** the geometric mean for bronchoalveolar lavage (BAL) samples are shown. Data graphed as the mean and SEM in **(A)** and **(C)** The p and R^2^ values are shown in **(B, D)**. The level of detection (LOD; dashed line) was 119 gene copies/mL (GC/mL) for sgRNA. For values below the LOD, half the LOD value (59 GC/mL) was used for graphing and calculation of the geometric mean. All statistical analyses performed using one-way ANOVA with Dunnett’s post-hoc comparison of vaccinated groups to placebo; where *p < 0.05, **p ≤ 0.01, and ****p ≤ 0.0001. For **(A, C)**, p values presented in **Supplementary Table S7**. **(E)** Individual (dashed lines) and group (solid line) NHP sera microneutralization 50 (MN50) are shown for Day 0 pre-challenge and Days 7 and 14 post-challenge. On Day 14, p = 0.0047 and = 0.0423 for SC > SC > Oral and SC > Oral > Oral groups compared to placebo, respectively.

The presence of replicating virus in nasal swab samples was determined by RT qPCR of subgenomic RNA (sgRNA). In nasal samples, sgRNA was below the LOD for two SC > SC > Oral NHP and two SC > Oral > Oral NHP on Day 3 and 4 post-challenge, respectively. sgRNA was below the LOD for all SC > Oral > Oral NHP by Day 5 and for all. SC > SC > Oral NHP by Day 7 post-challenge (**Figure 6A**, **Supplementary Table S5**). The geometric mean for nasal sgRNA (**Figure 6B**) allows visualization of the difference in decreases of sgRNA between vaccinated and placebo NHP over 7 days post-challenge.

Just one day after challenge, the sgRNA in lung (BAL samples) was significantly lower in vaccinated as compared to placebo NHP (**Figure 6C**, **Supplementary Table S6**) and by 7 days after challenge, sgRNA of 9/10 vaccinated NHP was below the LOD. The geometric mean (**Figure 6D**) reveals a difference of 2 logs between the vaccinated and placebo NHP on Day 1, suggesting a rapid clearance of replicating virus from the lungs within the first day.

No increases in sgRNA were detected at any time point.

For sera collected in the post-challenge period, a live virus-based microneutralization assay was used to assess SARS-CoV-2 neutralization capability as reflected by the ‘MN50’, that is, a ≥ 50% reduction in signal from the virus only to the cell control using the formula shown in *Methods*. An increase in neutralization capability of sera was only observed for vaccinated and not placebo NHP (**Figure 6E**). Further increases in vaccinated NHP sera neutralization capability were demonstrated at Day 14 post-challenge, at which time point the increases were significantly greater than the placebo group.

## Discussion

The study findings reported herein demonstrate that in the rhesus macaque NHP model, SC prime vaccination with two oral boosts with the dual-antigen hAd5 S-Fusion + N-ETSD vaccine provides protection from SARS-CoV-2 infection for both nasal and lung airways, as reflected by decreases in sgRNA, against SARS-CoV-2 challenge that is comparable to that observed with an SC prime followed by an SC and oral boost.

While there has been at least one report suggesting sgRNA may not necessarily represent replicating virus in clinical samples from patients with suspected infection (56), in a challenge study, where the timing of infection is known and controlled, the assumption can reasonably be made that sgRNA does indeed reflect the presence of replicating virus (57).

The ability of hAd5 S-Fusion + N-ETSD vaccination to elicit anti-S antibodies that were demonstrated to be neutralizing for 8 of 10 vaccinated NHP in the surrogate cPass neutralization assay (**Figure 3**) taken together with the rapid increase in sera neutralization capability as detected by the microneutralization assay post-challenge suggests the establishment of memory B cells by vaccination that were activated to produce large amounts of neutralizing antibodies post-challenge. This is a hypothesis that awaits further study. We note that, in our hands, the cPass assay was more sensitive to smaller differences in the pre-challenge sera samples, whereas the microneutralization assay better detected differences in neutralization capability in the post-challenge samples containing high levels of neutralizing antibodies.

A variety of other reports exist on vaccine testing in NHP, including an Ad26-vectored S vaccine (17), the mRNA1273 vaccine (58), ChAdOx1 (18), and a series of prototype DNA vaccines (59). All reported the generation of neutralizing anti-S antibodies and most activation of T cells, with the latter reflecting significant variation in individual animals, as seen in the present study. In these other studies, increases in nasal sgRNA post-challenge were detected for most vaccines/regimens, with the exception of high-dose mRNA-1273, before levels decreased. Overall, the titers of SARS-CoV-2 used for challenge were lower than that used here. The apparent near-immediate reduction of viral replication in nasal passages by hAd5 S-Fusion + N-ETSD vaccination taken together with the finding of rapid clearance in the lung, provide evidence that vaccination may have provided more than partial protection and may reduce or prevent transmission. A transmission model will be used in future studies to confirm this hypothesis.

We did not determine the immune correlates of protection in the present study, including the contribution of T cells to protection in the post-challenge period. We have initiated studies to assess these correlations and discern the contribution of N as well as the benefit of the ETSD modification as compared to unmodified N to enhancement of MHC II responses and protection as compared to S-only in the golden hamster challenge model (60).

The protection conferred by hAd5 S-Fusion + N-ETSD vaccination of NHPs by SC and oral boost administration particularly reveal the potential for this vaccine to be developed for world-wide distribution. The oral hAd5 S-Fusion + N-ETSD formulation is thermally stable (61) and does not require ultra-cold storage, which can be a challenge in remote or under-developed regions, like many COVID vaccines currently in development.

Oral, rather than injected, boosts further facilitate accessibility. An oral boost provides several advantages in SARS-CoV-2 vaccination, including a greater potential for generating mucosal immunity (62–64). SARS-CoV-2 is a mucosal virus (65, 66) and is only rarely detected in blood (67, 68), therefore vaccines that specifically target mucosal immunity are of interest (69). In future studies of our oral formulation, we will determine levels of IgA as part of our assessment of mucosal immunity.

Our thermally-stable oral hAd5 S-Fusion + N-ETSD vaccine, due its expression of S and N, also has the potential to act as a ‘universal’ boost to other previously administered vaccines that deliver only S antigens.

The hAd5 S-Fusion + N-ETSD vaccine delivered as an SC prime and boost is in Phase 1 clinical trials and the thermally-stable oral vaccine has entered Phase 1 trials as both a prime and boost, and as a boost to an SC prime.

## Data Availability Statement

The datasets presented in this study can be found in online repositories. The names of the repository/repositories and accession number(s) can be found in the article/**Supplementary Material**.

## Ethics Statement

The animal study was reviewed and approved by Battelle Institutional Animal Care and Use Committee (IACUC).

## Author Contributions

EG co-designed the vaccine vector and analyzed data. JS co-designed the NHP study, reviewed data and edited the manuscript. MV, AR, ABez, PSi, LZ, AS, BM, HA, AB, KD, JB, RW, HG, and PL performed assays and analyzed data. VP formulated the oral vaccine. ABac, PB, and JD created the thermally stable final oral formulation. DS supervised the NHP study. PSp analyzed data, generated graphs/figures and wrote the manuscript. LS helped develop the vaccine and edited the manuscript. SR co-developed the vaccine and edited the manuscript. KN co-designed the vaccine, supervised all analyses. PS-S co-designed, developed the vaccine, reviewed all data and co-wrote the manuscript. All authors contributed to the article and approved the submitted version.

## Funding

The NHP study performed at the Battelle Biomedical Research Center was sponsored by the Biomedical Advanced Research and Development Authority (BARDA), Office of the Assistant Secretary of Preparedness and Response (ASPR), Department of Health and Human Services (DHHS) and the National Institutes of Health/National Institute for Allergy and Infectious Diseases (NIH/NIAID), Washington, D.C. All other aspects of vaccine development and analyses were funded by ImmunityBio, Inc.

## Conflict of Interest

Authors EG, MU, AR, JS, PS, LZ, ABez, AS, BM, HA, RW, KD, JB, VP, HG, PL, PS, SR, KN and PS-S were employed by company ImmunityBio, Inc. Author LS was employed by company NantKwest, Inc. Authors ABac, PB and JD were employed by company IosBio.

The authors declare that this study received funding from ImmunityBio, Inc. The funder had the following involvement in the study: design and development of the vaccine and all NHP study-related analyses.

## Publisher’s Note

All claims expressed in this article are solely those of the authors and do not necessarily represent those of their affiliated organizations, or those of the publisher, the editors and the reviewers. Any product that may be evaluated in this article, or claim that may be made by its manufacturer, is not guaranteed or endorsed by the publisher.
