# Supplementary material for: Dual-Antigen COVID-19 Vaccine Subcutaneous Prime Delivery With Oral Boosts Protects NHP Against SARS-CoV-2 Challenge
Source: Front Immunol. 2021 Sep 16;12:729837. doi: 10.3389/fimmu.2021.729837 (PMC8481919; doi:10.3389/fimmu.2021.729837)
Supplement: Supplementary file 1 [file DataSheet_1.pdf]

Supplementary Material for:

Dual-antigen COVID-19 vaccine subcutaneous prime delivery with oral boosts protects NHP against SARS-CoV-2 challenge

Elizabeth Gabitzsch, Jeffrey T. Safrit, Mohit Verma, Adrian Rice, Peter Sieling, Lise Zakin, Annie Shin, Brett Morimoto, Helty Adisetiyo, Raymond Wong, Ashish Bezawada, Kyle Dinkins, Joseph Balint, Victor Peykov, Hermes Garban, Philip Liu, Andrew Bacon, Pete Bone, Jeff Drew, Daniel C. Sanford, Patricia Spilman, Lennie Sender, Shahrooz Rabizadeh, Kayvan Niazi, and Patrick Soon-Shiong

Vaccine construct sequences

The spike sequence used was based on Genbank accession number MN908947 (https://www.ncbi.nlm.nih.gov/nuccore/MN908947). The sequence used for 'S-fusion' is shown below with the HA tag underlined and the proprietary fusion sequence (immediately following HA) represented as Xs.

MFVFLVLLPLVSS<u>YPYDVPDYA</u>XXXXXXXXXXXXXXXXQCVNLTTRTQLPPAYTNSFT
RGVYYPDKVFRSSVLHSTQDLFLPFFSNVTWFHAIHVSGTNGTKRFDNPVLPFNDGVYF
ASTEKSNIIRGWIFGTTLDSKTQSLLIVNNATNVVIKVCEFQFCNDPFLGVYYHKNNKSW
MESEFRVYSSANNCTFEYVSQPFLMDLEGKQGNFKNLREFVFKNIDGYFKIYSKHTPINL
VRDLPQGFSALEPLVDLPIGINITRFQTLLALHRSYLTPGDSSSGWTAGAAAYYVGYLQP
RTFLLKYNENGTITDAVDCALDPLSETKCTLKSFTVEKGIYQTSNFRVQPTESIVRFPNIT
NLCPFGEVFNATRFASVYAWNRKRISNCVADYSVLYNSASFSTFKCYGVSPTKLNDLCF
TNVYADSFVIRGDEVRQIAPGQTGKIADYNYKLPDDFTGCVIAWNSNNLDSKVGGNYN
YLYRLFRKSNLKPFERDISTEIYQAGSTPCNGVEGFNCYFPLQSYGFQPTNGVGYQPYRV
VVLSFELLHAPATVCGPKKSTNLVKNKCVNFNFNGLTGTGVLTESNKKFLPFQQFGRDI
ADTTDAVRDPQTLEILDITPCSFGGVSVITPGTNTSNQVAVLYQDVNCTEVPVAIHADQL
TPTWRVYSTGSNVFQTRAGCLIGAEHVNNSYECDIPIGAGICASYQTQTNSPRRARSVAS
QSIIAYTMSLGAENSVAYSNNSIAIPTNFTISVTTEILPVSMTKTSVDCTMYICGDSTECSN
LLLQYGSFCTQLNRALTGIAVEQDKNTQEVFAQVKQIYKTPPIKDFGGFNFSQILPDPSKP
SKRSFIEDLLFNKVTLADAGFIKQYGDCLGDIAARDLICAQKFNGLTVLPPLLTDEMIAQ
YTSALLAGTITSGWTFGAGAALQIPFAMQMAYRFNGIGVTQNVLYENQKLIANQFNSAI
GKIQDSLSSTASALGKLQDVVNQNAQALNTLVKQLSSNFGAISSVLNDILSRLDKVEAEV
QIDRLITGRLQSLQTYVTQQLIRAAEIRASANLAATKMSECVLGQSKRVDFCGKGYHLM
SFPQSAPHGVVFLHVTYVPAQEKNFTTAPAICHDGKAHFPREGVFVSNGTHWFVTQRNF
YEPQIITTDNTFVSGNCDVVIGIVNNTVYDPLQPELDSFKEELDKYFKNHTSPDVDLGDIS
GINASVVNIQKEIDRLNEVAKNLNESLIDLQELGKYEQYIKWPWYIWLGFIAGLIAIVMV
TIMLCCMTSCCSCLKGCCSCGSCCKFDEDDSEPVLKGVKLHYT

The N sequence used was based on GenBank accession number MN908947 (https://www.ncbi.nlm.nih.gov/nuccore/MN908947). The proprietary ETSD sequences (both 3' and 5') are shown as Os, and the FLAG tag immediately following is underlined; linkers bracketing the NLV peptide (the first 3 amino acids of 9 amino acid CMV phosphoprotein 65, used to evaluate non-vaccine antigen-elicited T cell responses) are highlighted in grey.

OOOOOOOOOOOOOOOOOOODYKDDDDKMSDNGPQNQRNAPRITFGGPSDSTGSNQN
GERSGARSKQRRPQGLPNNTASWFTALTQHGKEDLKFPRGQGVPINTNSSPDDQIGYYR
RATRRIRGGDGKMKDLSPRWYFYYLGTGPEAGLPYGANKDGIIWVATEGALNTPKDHI
GTRNPANNAAIVLQLPQGTTLPKGFYAEGSRGGSQASSRSSSRSRNSSRNSTPGSSRGTSP
ARMAGNGGDAALALLLLDRLNQLESKMSGKGQQQQGQTVTKKSAAEASKKPRQKRT
ATKAYNVTQAFGRRGPEQTQGNFGDQELIRQGTDYKHWPQIAQFAPSASAFFGMSRIG
MEVTPSGTWLTYTGAIKLDDKDPNFKDQVILLNKHIDAYKTFPPTEPKKDKKKKADETQ
ALPQRQKKQQTVTLLPAADLDDFSKQLQQSMSSADSTQAGPGPGNLVPMVATVGPGPG
OOOOOOOOOOOOOOOOOOOOOOOOOOOOOOOOOO

The NHP Study

Clinical signs

No clinical signs were noted during the twice daily observations for clinical signs of toxicity due to vaccination and no animals died during the two weeks after one subcutaneous immunization of 1x 10^{11} VP or a week after an oral booster of 1x10^{10} IU of hAd5-S-Fusion+N-ETSD. No gross pathological effect or adverse events were observed and there were no notable changes in body weight (Supplementary Fig. S1).

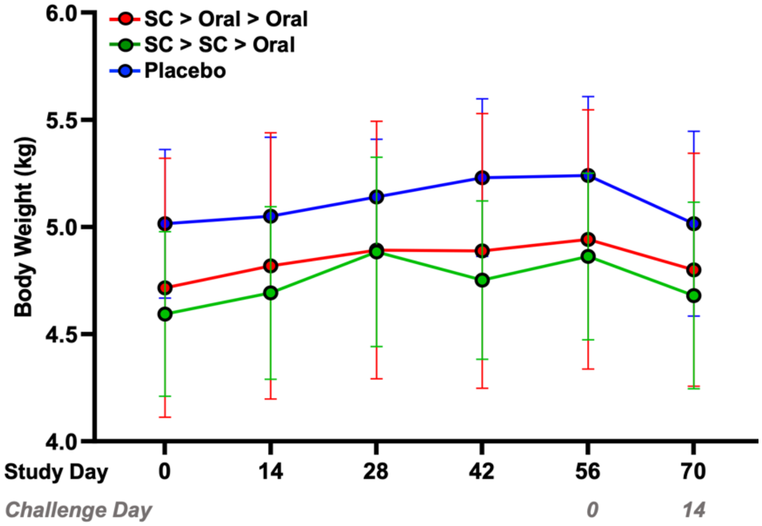

Figure S1. *NHP body weights over the course of the study*. Body weights are shown in Kg; note y-axis range is 4-6 Kg.

Hematology

Hematological parameters evaluated in Group 1 and 2 hAd5 S-Fusion + N-ETSD and Group 3 placebo control animals are presented in Table S1**.** Only hemoglobin was consistently slightly lower in Group 2 NHP as compared to Group 3 placebo for all post-vaccination time points.

Table S1. *Hematological parameters in hAd5 S-Fusion + N-ETSD vaccinated Group 1 and Group 2 NHP versus placebo Group 3*.

Study Day	Day 0			Day 14			Day 28			Day 42			Day 56			Day 63			Day 70		
**Parameter**	**G1**	**G2**	**G3**	**G1**	**G2**	**G3**	**G1**	**G2**	**G3**	**G1**	**G2**	**G3**	**G1**	**G2**	**G3**	**G1**	**G2**	**G3**	**G1**	**G2**	**G3**
White Blood Cell Count	ND	ND	-	ND	ND	-	ND	ND	-	ND	ND	-	ND	ND	-	ND	ND	-	ND	ND	-
Red Blood Cell Count	ND	ND	-	ND	ND	-	5.42	↓4.88	5.42	ND	ND	-	ND	ND	-	ND	ND	-	ND	ND	-
Hemoglobin	ND	ND	-	13.26	↓11.18	13.10	13.84	↓11.80	13.90	12.90	↓11.24	12.75	13.88	↓12.00	14.05	12.42	↓10.36	11.95	13.86	↓11.74	13.10
Hematocrit	ND	ND	-	40.68	34.78	41.30	42.70	↓36.88	45.00	ND	ND	-	ND	ND	-	ND	ND	-	ND	ND	-
MCV	ND	ND	-	ND	ND	-	ND	ND	-	ND	ND	-	ND	ND	-	ND	ND	-	ND	ND	-
MCH	ND	ND	-	ND	ND	-	ND	ND	-	ND	ND	-	ND	ND	-	ND	ND	-	ND	ND	-
MCHC	ND	ND	-	ND	ND	-	ND	ND	-	ND	ND	-	ND	ND	-	ND	ND	-	ND	ND	-
CHCM	ND	ND	-	ND	ND	-	ND	ND	-	ND	ND	-	ND	ND	-	ND	ND	-	ND	ND	-
RDW	ND	ND	-	ND	ND	-	ND	ND	-	ND	ND	-	ND	ND	-	ND	ND	-	ND	ND	-
Platelet Count	305.0	↑424.4	293.5	ND	ND	-	ND	ND	-	ND	ND	-	ND	ND	-	ND	ND	-	ND	ND	-
MPV	ND	ND	-	ND	ND	-	ND	ND	-	ND	ND	-	ND	ND	-	ND	ND	-	ND	ND	-
%NEUT	ND	ND	-	ND	ND	-	ND	ND	-	ND	ND	-	ND	ND	-	ND	ND	-	ND	ND	-
%LYMPH	ND	ND	-	ND	ND	-	ND	ND	-	ND	ND	-	ND	ND	-	ND	ND	-	ND	ND	-
%MONO	ND	ND	-	ND	ND	-	ND	ND	-	ND	ND	-	ND	ND	-	ND	ND	-	ND	ND	-
%EOS	ND	ND	-	ND	ND	-	ND	ND	-	ND	ND	-	ND	ND	-	ND	ND	-	ND	ND	-
%BASO	0.12	↑ 0.22	0.10	ND	ND	-	ND	ND	-	ND	ND	-	↓ 1.06	1.14	2.05	ND	ND	-	ND	ND	-
%LUC	ND	ND	-	ND	ND	-	ND	ND	-	ND	ND	-	ND	ND	-	ND	ND	-	ND	ND	-
#NEUT	ND	ND	-	ND	ND	-	ND	ND	-	ND	ND	-	ND	ND	-	ND	ND	-	ND	ND	-
#LYMPH	ND	ND	-	ND	ND	-	ND	ND	-	ND	ND	-	ND	ND	-	ND	ND	-	ND	ND	-
#MONO	ND	ND	-	ND	ND	-	ND	ND	-	ND	ND	-	ND	ND	-	ND	ND	-	ND	ND	-
#EOS	ND	ND	-	ND	ND	-	ND	ND	-	ND	ND	-	ND	ND	-	ND	ND	-	ND	ND	-
#BASO	ND	ND	-	ND	ND	-	ND	ND	-	ND	ND	-	ND	ND	-	ND	ND	-	ND	ND	-
#LUC	ND	ND	-	ND	ND	-	ND	ND	-	ND	ND	-	ND	ND	-	ND	ND	-	ND	ND	-
Neut%/Lymph% Ratio	ND	ND	-	ND	ND	-	ND	ND	-	ND	ND	-	ND	ND	-	ND	ND	-	ND	ND	-
Neut#/Lymph# Ratio	ND	ND	-	ND	ND	-	ND	ND	-	ND	ND	-	ND	ND	-	ND	ND	-	ND	ND	-

ND= Difference between placebo control and hAd5 S-Fusion + N-ETSD vaccinated NHP for this time point was not statistically significant
↑= increase was statistically significant ($p < 0.05$)
↓= decrease was statistically significant ($p < 0.05$)

Clinical Chemistry

Only creatinine was consistently lower in Group 1 NHP on five time points and at two time points for Group 2 (Table S2), but it was lower on Day 0 baseline, indicating this was not vaccine-related.

Table S2. *Clinical chemistry for hAd5 S-Fusion + N-ETSD vaccinated Group 1 and Group 2 NHP as compared to placebo Group 3.* ND – not significantly different from placebo. Statistical analyses performed using One-way ANOVA.

Study Day	Day 0			Day 14			Day 28			Day 42			Day 56			Day 63			Day 70		
**Parameter**	**G1**	**G2**	**G3**	**G1**	**G2**	**G3**	**G1**	**G2**	**G3**	**G1**	**G2**	**G3**	**G1**	**G2**	**G3**	**G1**	**G2**	**G3**	**G1**	**G2**	**G3**
Glucose	ND	ND	-	ND	ND	-	ND	ND	-	ND	ND	-	ND	ND	-	ND	ND	-	ND	ND	-
Blood Urea Nitrogen	ND	ND	-	ND	ND	-	ND	ND	-	ND	ND	-	ND	ND	-	ND	ND	-	ND	ND	-
Creatinine	↓0.57	0.66	0.79	↓0.49	0.58	0.75	↓0.69	0.76	0.88	↓0.59	↓0.64	0.82	↓0.67	↓0.71	0.88	ND	ND	-	ND	ND	-
BUN/Creatinine Ratio	ND	ND	-	32.74	↑42.98	27.15	ND	ND	-	ND	ND	-	ND	ND	-	ND	ND	-	ND	ND	-
Sodium	ND	ND	-	ND	ND	-	ND	ND	-	ND	ND	-	ND	ND	-	ND	ND	-	ND	ND	-
Potassium	ND	ND		ND	ND	-	ND	ND	-	ND	ND	-	ND	ND	-	ND	ND	-	ND	ND	-
Chloride	ND	ND	-	ND	ND	-	ND	ND	-	ND	ND	-	ND	ND	-	ND	ND	-	ND	ND	-
Calcium	ND	ND	-	9.50	↓9.17	9.78	ND	ND	-	ND	ND	-	ND	ND	-	ND	ND	-	ND	ND	-
Phosphorus	ND	ND	-	ND	ND	-	ND	ND	-	ND	ND	-	ND	ND	-	ND	ND	-	ND	ND	-
Total Bilirubin	ND	ND	-	ND	ND	-	ND	ND	-	ND	ND	-	ND	ND	-	ND	ND	-	ND	ND	-
Total Protein	ND	ND	-	ND	ND	-	ND	ND	-	ND	ND	-	ND	ND	-	ND	ND	-	ND	ND	-
Albumin	ND	ND	-	4.40	↓4.00	4.67	ND	ND	-	ND	ND	-	ND	ND	-	ND	ND	-	ND	ND	-
Globulin	ND	ND	-	ND	ND	-	ND	ND	-	ND	ND	-	ND	ND	-	ND	ND	-	ND	ND	-
A/G Ration	ND	ND	-	ND	ND	-	ND	ND	-	ND	ND	-	ND	ND	-	ND	ND	-	ND	ND	-
Alanine Aminotransferase	50.42	↓31.60	55.60	ND	ND	-	ND	ND	-	ND	ND	-	ND	ND	-	ND	ND	-	ND	ND	-
Aspartate Aminotransferase	ND	ND	-	ND	ND	-	ND	ND	-	ND	ND	-	ND	ND	-	ND	ND	-	ND	ND	-
Alkaline Phosphatase	ND	ND	-	ND	ND	-	ND	ND	-	ND	ND	-	ND	ND	-	ND	ND	-	ND	ND	-
Lactate Dehydrogenase	ND	ND	-	ND	ND	-	ND	ND	-	ND	ND	-	ND	ND	-	ND	ND	-	ND	ND	-
Gamma Glutamyl Transferase	ND	ND	-	ND	ND	-	ND	ND	-	ND	ND	-	ND	ND	-	ND	ND	-	ND	ND	-
Sorbitol Dehydrogenase	ND	ND	-	ND	ND	-	ND	ND	-	ND	ND	-	ND	ND	-	ND	ND	-	ND	ND	-
C-Reactive Protein	ND	ND	-	ND	ND	-	ND	ND	-	ND	ND	-	ND	ND	-	ND	ND	-	ND	ND	-

ND= Difference between placebo control and hAd5 S-Fusion + N-ETSD vaccinate NHP for this time point was not statistically significant
↑= increase was statistically significant ($p < 0.05$)
↓= decrease was statistically significant ($p < 0.05$)

Viral gRNA in nasal swab samples

Viral load was determined in nasal swab samples by RT qPCR of genomic RNA. The data for individual NHP at time point post-challenge are shown in **Table S3**.

Table S3. *Gene copies per mL for gRNA as determined by RT qPCR in nasal passages for all groups.* Values below the level of detection (LOD) of 54 gene copies per mL (GC/mL) are highlighted in yellow. For calculation of the geometric mean, half the LOD values (27 GC/mL) was used.

Animal ID	Grp #	Routes	Sex	Day 56	Day 57	Day 58	Day 59	Day 60	Day 61	Day 62	Day 63	Day 70
RA3938	1	SC-SC-Oral	Male	<LOD	5.47E+08	3.67E+06	2.52E+04	2.50E+04	2.90E+03	1.87E+02	1.04E+03	<LOD
RA3946	1	SC-SC-Oral	Male	<LOD	2.59E+08	2.04E+07	1.38E+06	2.46E+05	9.01E+04	3.29E+03	<LOD	<LOD
RA3945	1	SC-SC-Oral	Male	<LOD	2.06E+07	1.19E+07	9.32E+05	1.73E+05	1.97E+03	9.83E+03	5.71E+02	4.29E+02
RA4002	1	SC-SC-Oral	Female	<LOD	3.84E+07	1.21E+07	5.80E+03	1.95E+05	9.95E+03	<LOD	<LOD	<LOD
RA4000	1	SC-SC-Oral	Female	<LOD	1.52E+10	1.66E+04	2.75E+06	9.18E+05	7.36E+04	1.53E+04	<LOD	7.79E+01
RA3936	2	SC-Oral-Oral	Male	<LOD	1.13E+08	5.06E+06	2.53E+06	5.02E+05	2.04E+03	1.50E+03	<LOD	<LOD
RA3942	2	SC-Oral-Oral	Male	<LOD	3.13E+08	3.59E+06	8.15E+05	5.62E+03	3.32E+02	<LOD	1.01E+02	<LOD
RA3999	2	SC-Oral-Oral	Female	<LOD	1.80E+08	2.65E+07	1.14E+06	3.31E+04	2.39E+03	1.41E+03	1.76E+02	<LOD
RA4014	2	SC-Oral-Oral	Female	<LOD	1.18E+09	4.64E+07	2.69E+05	3.09E+04	1.59E+03	1.68E+03	1.18E+02	<LOD
RA4001	2	SC-Oral-Oral	Female	<LOD	1.67E+07	4.87E+06	3.89E+05	3.95E+04	5.48E+03	1.90E+03	<LOD	2.74E+02
RA3949	3	SC-Oral-Oral	Male	<LOD	2.06E+09	4.95E+08	4.12E+06	7.14E+07	7.02E+05	2.73E+05	1.62E+04	1.74E+04
RA4011	3	SC-Oral-Oral	Female	<LOD	6.95E+07	2.38E+07	3.00E+07	3.44E+05	1.56E+05	7.19E+05	4.61E+05	1.47E+04

Viral gRNA in bronchoalveolar lavage (BAL) samples

Viral load was also determined in BAL samples. Gene copies per mL for individual NHP are shown in **Table S4.**

Table S4. *Gene copies per mL for gRNA as determined by RT qPCR in BAL samples for all groups.* Values below the level of detection (LOD) of 54 gene copies per mL (GC/mL) are highlighted in yellow. For calculation of the geometric mean, half the LOD values (27 GC/mL) was used.

Animal ID	Group	Routes	Sex	Day 42	Day 57	Day 59	Day 61	Day 63
RA3938	1	SC-SC-Oral	Male	<LOD	1.02E+06	1.26E+05	<LOD	5.24E+02
RA3946	1	SC-SC-Oral	Male	<LOD	1.25E+05	1.04E+05	5.43E+04	3.31E+02
RA3945	1	SC-SC-Oral	Male	<LOD	1.33E+05	7.50E+04	1.22E+05	3.54E+03
RA4002	1	SC-SC-Oral	Female	<LOD	1.40E+06	1.59E+04	8.74E+03	1.55E+02
RA4000	1	SC-SC-Oral	Female	<LOD	1.44E+05	5.00E+03	<LOD	<LOD
RA3936	2	SC-Oral-Oral	Male	<LOD	1.42E+06	3.55E+04	8.53E+03	1.43E+02
RA3942	2	SC-Oral-Oral	Male	<LOD	4.80E+04	1.40E+02	<LOD	1.75E+02
RA3999	2	SC-Oral-Oral	Female	<LOD	3.00E+06	2.28E+06	1.06E+06	1.31E+02
RA4014	2	SC-Oral-Oral	Female	<LOD	2.41E+06	7.39E+05	2.97E+04	1.90E+02
RA4001	2	SC-Oral-Oral	Female	<LOD	6.64E+03	<LOD	<LOD	<LOD
RA3949	3	SC-Oral-Oral	Male	<LOD	6.11E+07	3.44E+06	1.16E+05	2.35E+04
RA4011	3	SC-Oral-Oral	Female	<LOD	1.05E+07	1.20E+06	3.36E+05	1.74E+04

Viral sgRNA in nasal swab samples

Gene copies per mL of subgenomic RNA (sgRNA) for individual NHP are shown in **Table S5.**

Table S5. *Data for sgRNA in nasal swab samples as determined by RT qPCR passages for all groups.* Values below the level of detection (LOD) of 119 gene copies per mL (GC/mL) are

highlighted in yellow. For calculation of the geometric mean, half the LOD values (59 GC/mL) was used.

Animal ID	Group	Routes	Sex	Day 56	Day 57	Day 58	Day 59	Day 60	Day 61	Day 62	Day 63	Day 70
RA3938	1	SC-SC-Oral	Male	<LOD	1.12E+07	1.27E+05	<LOD	3.03E+02	<LOD	<LOD	<LOD	<LOD
RA3946	1	SC-SC-Oral	Male	<LOD	1.87E+07	1.21E+06	1.49E+05	1.32E+04	1.64E+04	<LOD	<LOD	<LOD
RA3945	1	SC-SC-Oral	Male	<LOD	1.81E+06	6.41E+05	6.41E+04	1.35E+04	<LOD	1.07E+03	<LOD	<LOD
RA4002	1	SC-SC-Oral	Female	<LOD	3.55E+06	6.65E+05	<LOD	1.45E+04	<LOD	<LOD	<LOD	<LOD
RA4000	1	SC-SC-Oral	Female	<LOD	2.73E+08	4.27E+03	2.38E+05	6.19E+04	<LOD	<LOD	<LOD	<LOD
RA3936	2	SC-Oral-Oral	Male	<LOD	6.57E+06	4.43E+05	1.71E+05	2.52E+04	<LOD	<LOD	<LOD	<LOD
RA3942	2	SC-Oral-Oral	Male	<LOD	1.58E+07	3.43E+05	1.12E+03	<LOD	<LOD	<LOD	<LOD	<LOD
RA3999	2	SC-Oral-Oral	Female	<LOD	1.81E+07	1.99E+06	1.16E+05	1.90E+03	<LOD	<LOD	<LOD	<LOD
RA4014	2	SC-Oral-Oral	Female	<LOD	3.33E+07	2.32E+06	3.26E+04	<LOD	<LOD	<LOD	<LOD	<LOD
RA4001	2	SC-Oral-Oral	Female	<LOD	1.42E+06	4.97E+05	3.84E+04	5.98E+02	<LOD	<LOD	<LOD	<LOD
RA3949	3	SC-Oral-Oral	Male	<LOD	1.33E+08	1.84E+07	3.21E+05	1.49E+06	1.23E+04	2.73E+03	2.86E+02	<LOD
RA4011	3	SC-Oral-Oral	Female	<LOD	4.47E+06	3.81E+06	2.48E+06	5.88E+04	4.40E+04	2.04E+05	1.56E+05	<LOD

Viral sgRNA in BAL samples

Gene copies per mL of subgenomic RNA (sgRNA) for individual NHP are shown in **Table S6**.

Table S6. *Data for sgRNA in BAL samples as determined by RT qPCR passages for all groups.* Values below the level of detection (LOD) of 119 gene copies per mL (GC/mL) are highlighted in yellow. For calculation of the geometric mean, half the LOD values (59 GC/mL) was used.

Animal ID	Group	Routes	Sex	Day 42	Day 57	Day 59	Day 61	Day 63
RA3938	1	SC-SC-Oral	Male	<LOD	9.84E+04	3.48E+04	<LOD	2.58E+02
RA3946	1	SC-SC-Oral	Male	<LOD	2.06E+04	1.12E+04	4.57E+03	<LOD
RA3945	1	SC-SC-Oral	Male	<LOD	1.58E+04	1.32E+04	1.94E+04	<LOD
RA4002	1	SC-SC-Oral	Female	<LOD	1.04E+05	3.11E+03	6.03E+03	<LOD
RA4000	1	SC-SC-Oral	Female	<LOD	9.63E+03	<LOD	<LOD	<LOD
RA3936	2	SC-Oral-Oral	Male	<LOD	1.56E+05	1.11E+04	1.31E+03	<LOD
RA3942	2	SC-Oral-Oral	Male	<LOD	8.88E+03	5.65E+02	<LOD	<LOD
RA3999	2	SC-Oral-Oral	Female	<LOD	2.81E+05	1.38E+05	1.47E+05	<LOD
RA4014	2	SC-Oral-Oral	Female	<LOD	2.74E+05	1.19E+05	1.24E+04	<LOD
RA4001	2	SC-Oral-Oral	Female	<LOD	1.32E+03	<LOD	<LOD	<LOD
RA3949	3	SC-Oral-Oral	Male	<LOD	1.91E+06	5.05E+05	1.57E+04	5.12E+03
RA4011	3	SC-Oral-Oral	Female	<LOD	6.78E+05	9.89E+04	8.58E+03	5.67E+03

P values for nasal and BAL gRNA and sgRNA vaccinated NHP compared with placebo

The p values representing statistically significant differences between vaccinated NHP and placebo control g RNA (Fig. 5) and sgRNA (Fig. 6) when compared with placebo are shown in **Table S7**.

Table S7. *The p values for statistically significant differences in gRNA and sgRNA for vaccinated versus placebo NHP.* P values are shown for nasal and BAL gRNA (Fig. 5) and sgRNA (Fig. 6) for differences that were statistically significance when vaccinated NHP sample results were compared to placebo using one-way ANOVA with Dunnett's post-hoc comparison.

Day Post-challenge	Nasal gRNA p value vs placebo		BAL gRNA p value vs placebo		Nasal sgRNA p value vs placebo		BAL sgRNA p value vs placebo	
	SC > SC > Oral	SC > Oral > Oral	SC > SC > Oral	SC > Oral > Oral	SC > SC > Oral	SC > Oral > Oral	SC > SC > Oral	SC > Oral > Oral
1			0.0110	0.0124			0.0015	0.0026
2	0.0420	0.0478			0.0097	0.0132		
3	0.0210	0.0210	0.0810		0.0233	0.0218	0.0167	0.0348
4					0.0240	0.0386		
5	0.0102	0.0064			0.0140	0.0075		
6	0.0006	0.0006			0.0469	0.0465		
7	0.0388	0.0367	< 0.0001	< 0.0001			<0.0001	<0.0001
Only values showing signficance shown								
